# Supplementary material for: Wave-based liquid-interface metamaterials
Source: Nat Commun. 2017 Feb 9;8:14325. doi: 10.1038/ncomms14325 (PMC5311468; doi:10.1038/ncomms14325)
Supplement: Supplementary Information — Supplementary Figures [file ncomms14325-s1.pdf]

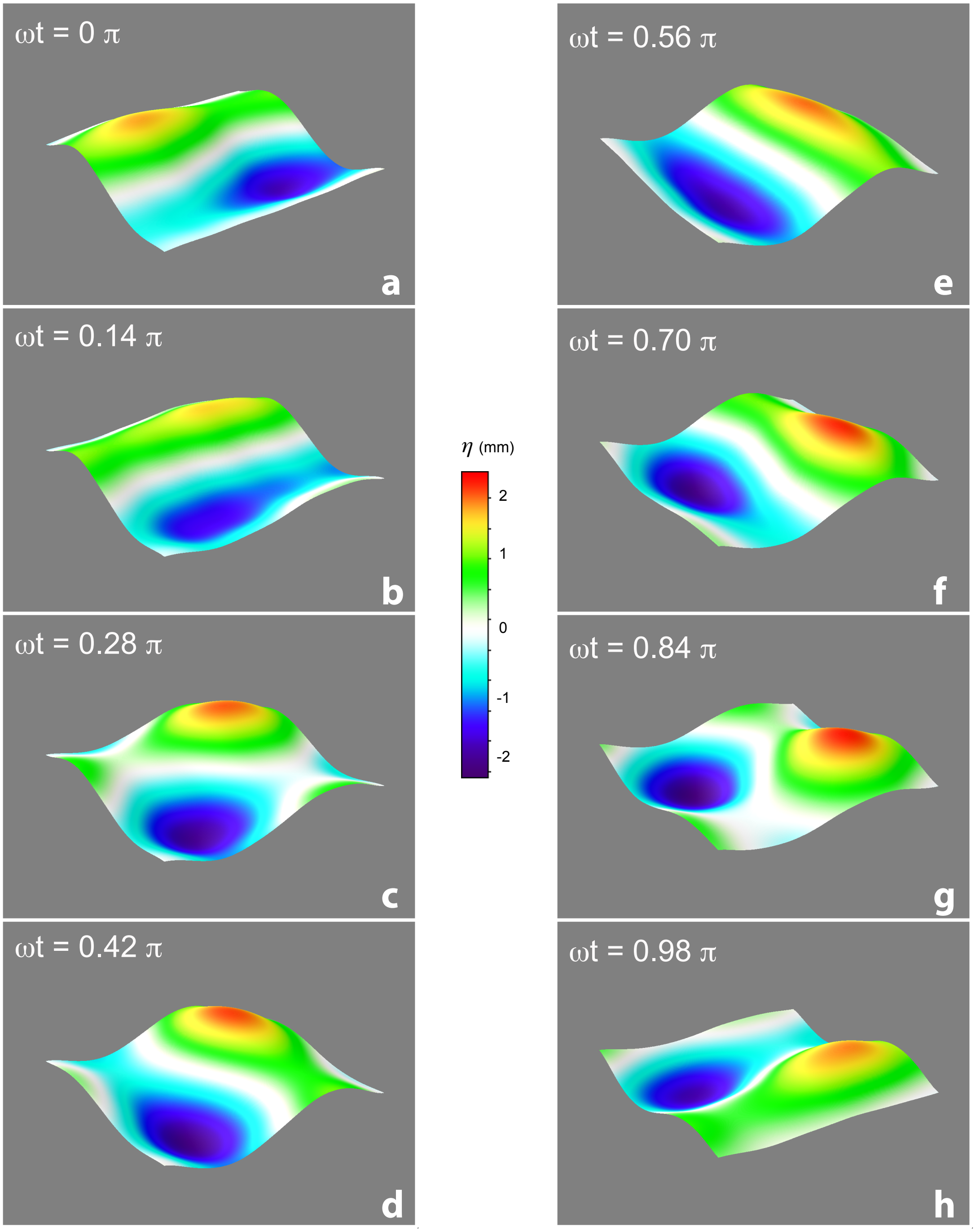
ωt = 0 π
a
ωt = 0.14 π
b
ωt = 0.28 π
c
ωt = 0.42 π
d
ωt = 0.56 π
e
ωt = 0.70 π
f
ωt = 0.84 π
g
ωt = 0.98 π
h
η (mm)
2
1
0
-1
-2

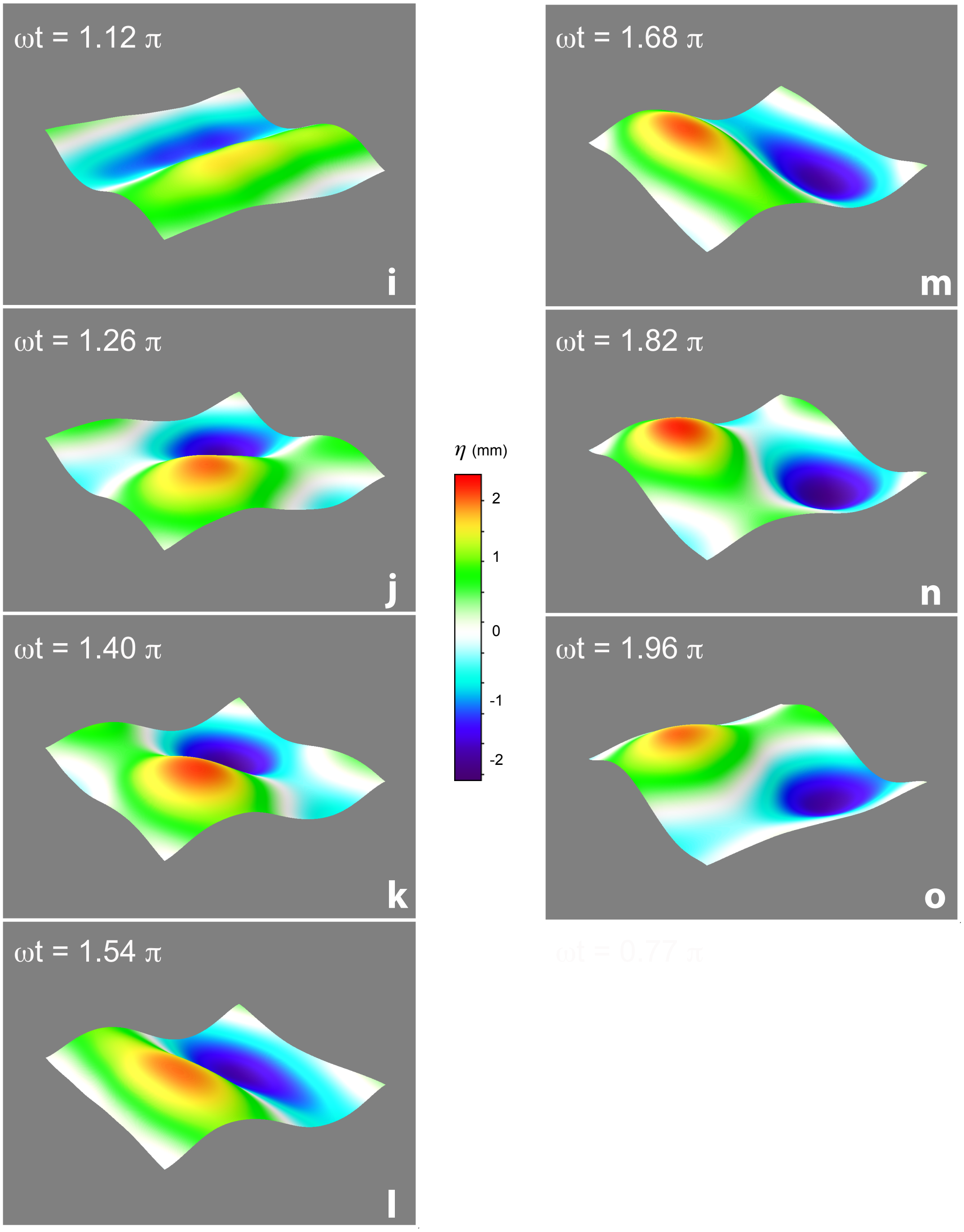

Supplementary Figure 1: Experimental measurements of the surface elevation. **a-o**: Experimental measurements of the wave field temporal evolution for two orthogonal standing surface waves phase shifted by $\phi = \pi/2$ within a $\lambda/2 \times \lambda/2$ box, i.e. a unit cell. (experimental parameters, $f = 2\pi/\omega = 3.9$ Hz, $\lambda = 104$ mm, $H = 2.5$ mm, movie acquired at 55 fps) (see Movie 2)

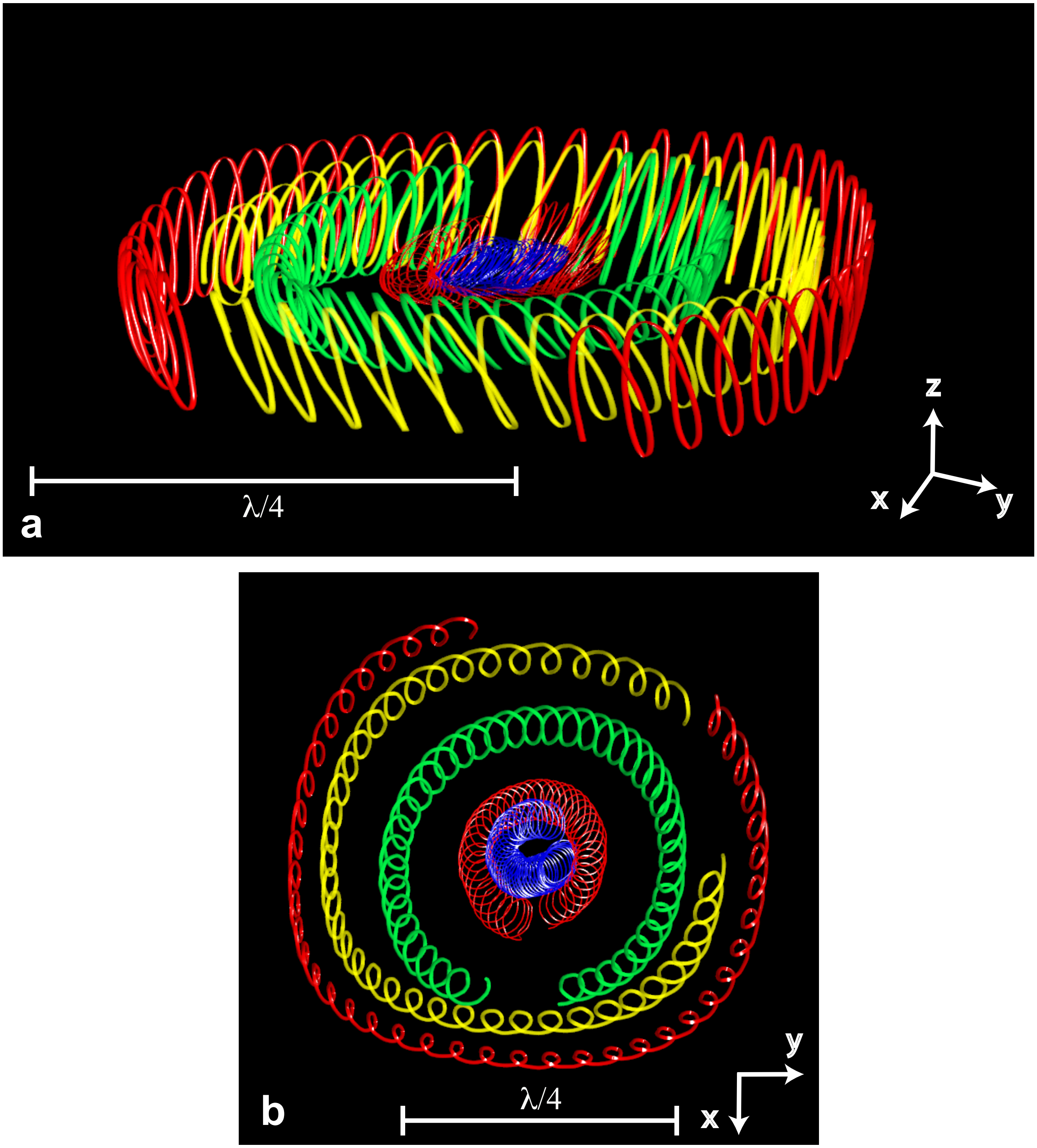

Supplementary Figure 2: Experimentally measured 3D trajectories of particles. Trajectories of particles drifting within a unit cell, **a** side view (perspective view), **b** top view (orthogonal projection). (experimental parameters, $f = 2\pi/\omega = 3.9$ Hz, $\lambda = 104$ mm, $H = 2.5$ mm, movie acquired at 55 fps).

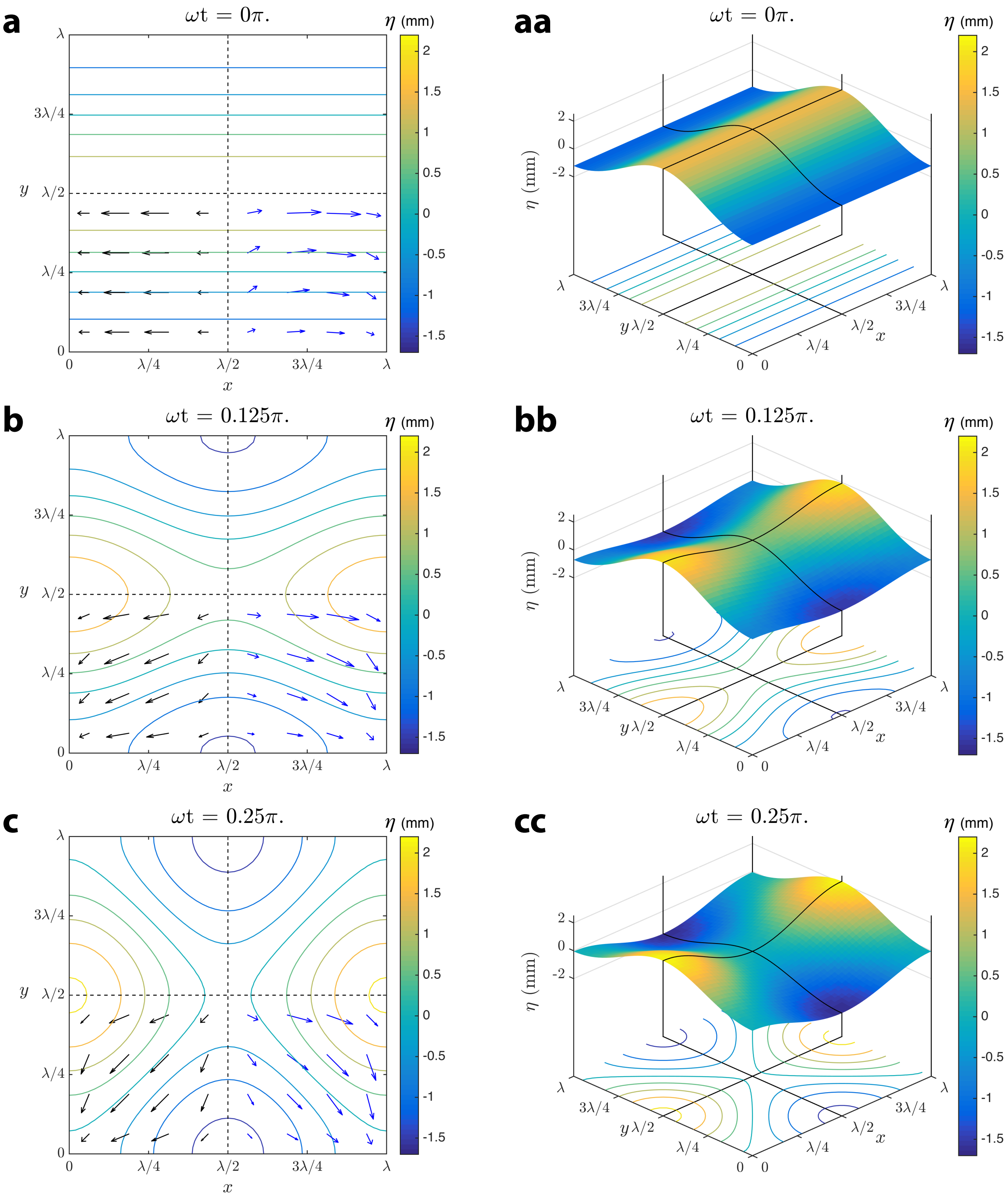
a
ωt = 0π.
η (mm)
aa
ωt = 0π.
η (mm)
b
ωt = 0.125π.
η (mm)
bb
ωt = 0.125π.
η (mm)
c
ωt = 0.25π.
η (mm)
cc
ωt = 0.25π.
η (mm)
x
y
0
λ/4
λ/2
3λ/4
λ
2
1.5
1
0.5
0
-0.5
-1
-1.5
η (mm)
2
0
-2

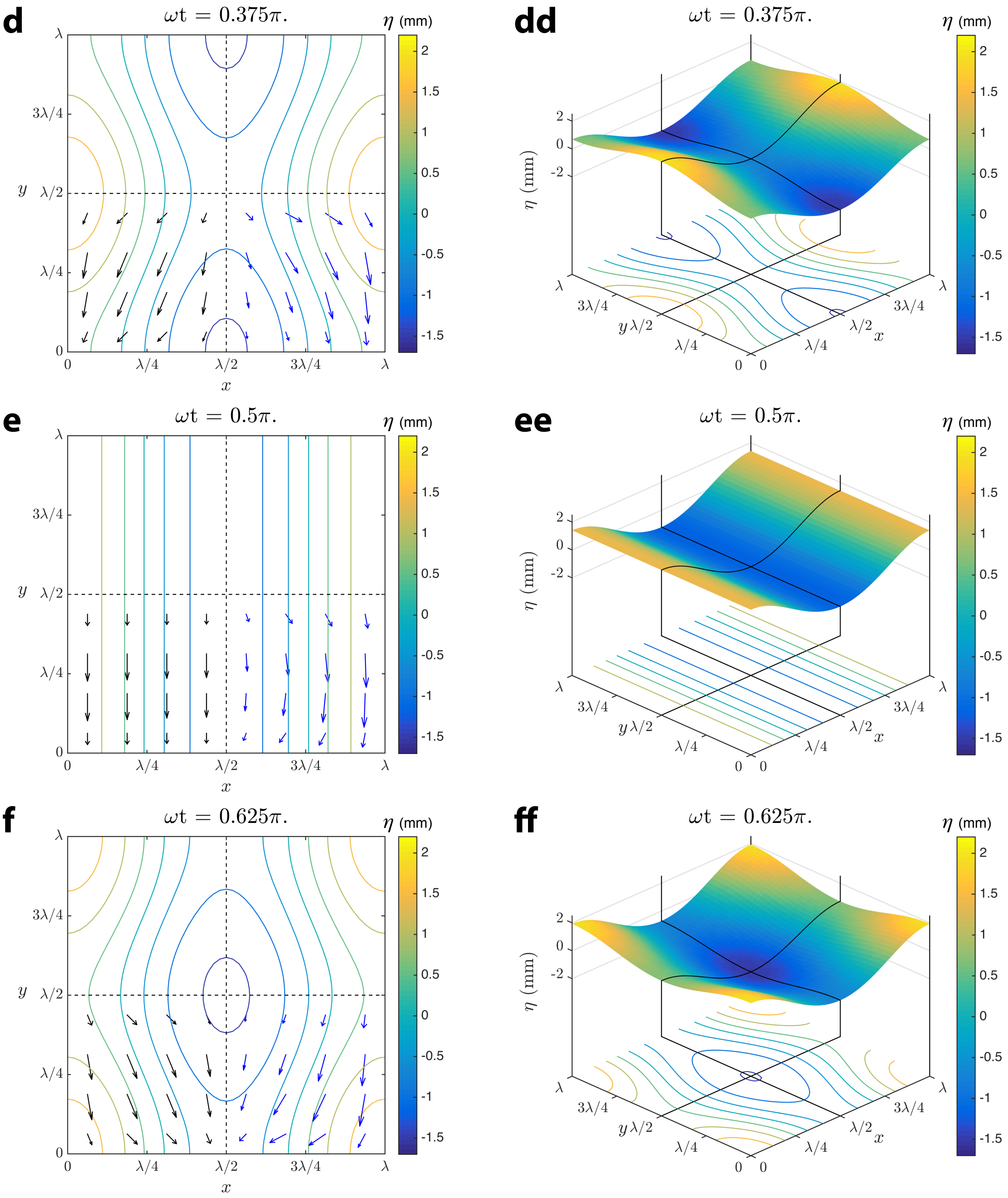
d
ωt = 0.375π.
η (mm)
dd
ωt = 0.375π.
η (mm)
e
ωt = 0.5π.
η (mm)
ee
ωt = 0.5π.
η (mm)
f
ωt = 0.625π.
η (mm)
ff
ωt = 0.625π.
η (mm)
x
y
η (mm)
0
λ/4
λ/2
3λ/4
λ
-1.5
-1
-0.5
0
0.5
1
1.5
2
-2

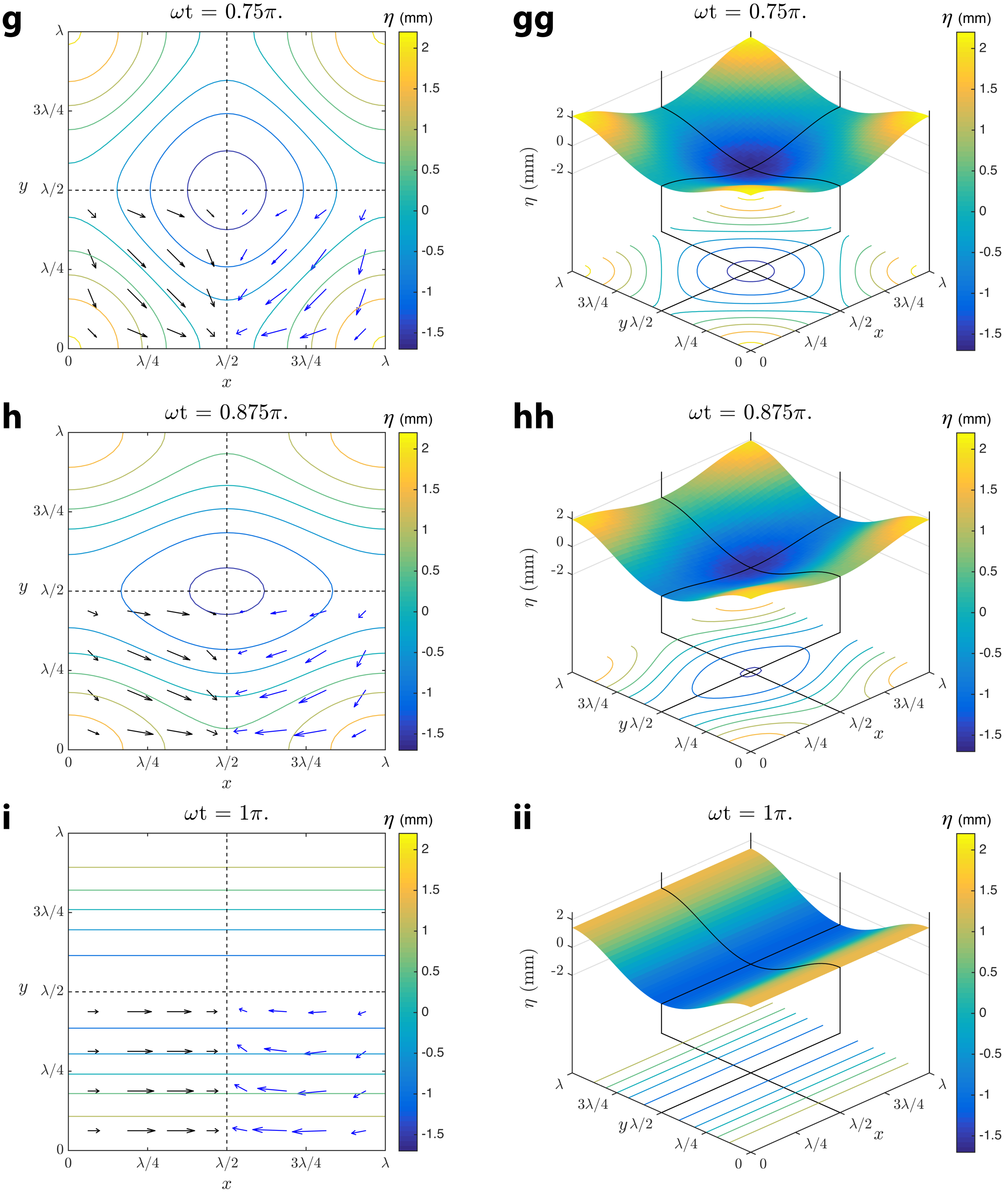

g
ωt = 0.75π.
η (mm)
gg
ωt = 0.75π.
η (mm)
h
ωt = 0.875π.
η (mm)
hh
ωt = 0.875π.
η (mm)
i
ωt = 1π.
η (mm)
ii
ωt = 1π.
η (mm)
x
y
0
λ/4
λ/2
3λ/4
λ
2
1.5
1
0.5
0
-0.5
-1
-1.5
-2

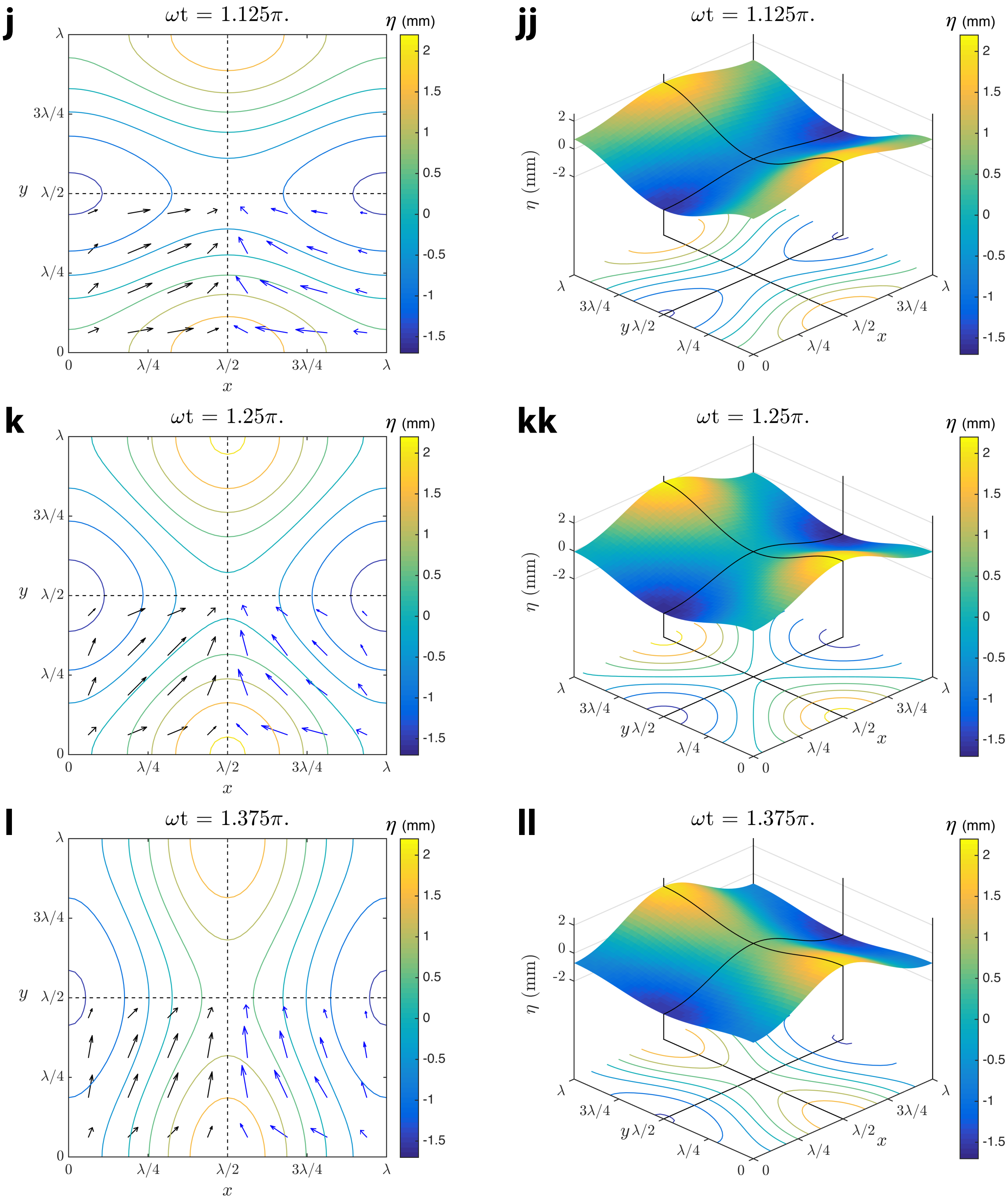
j
ωt = 1.125π.
η (mm)
jj
ωt = 1.125π.
k
ωt = 1.25π.
kk
ωt = 1.25π.
l
ωt = 1.375π.
ll
ωt = 1.375π.
x
y
λ/4
λ/2
3λ/4
λ
η (mm)

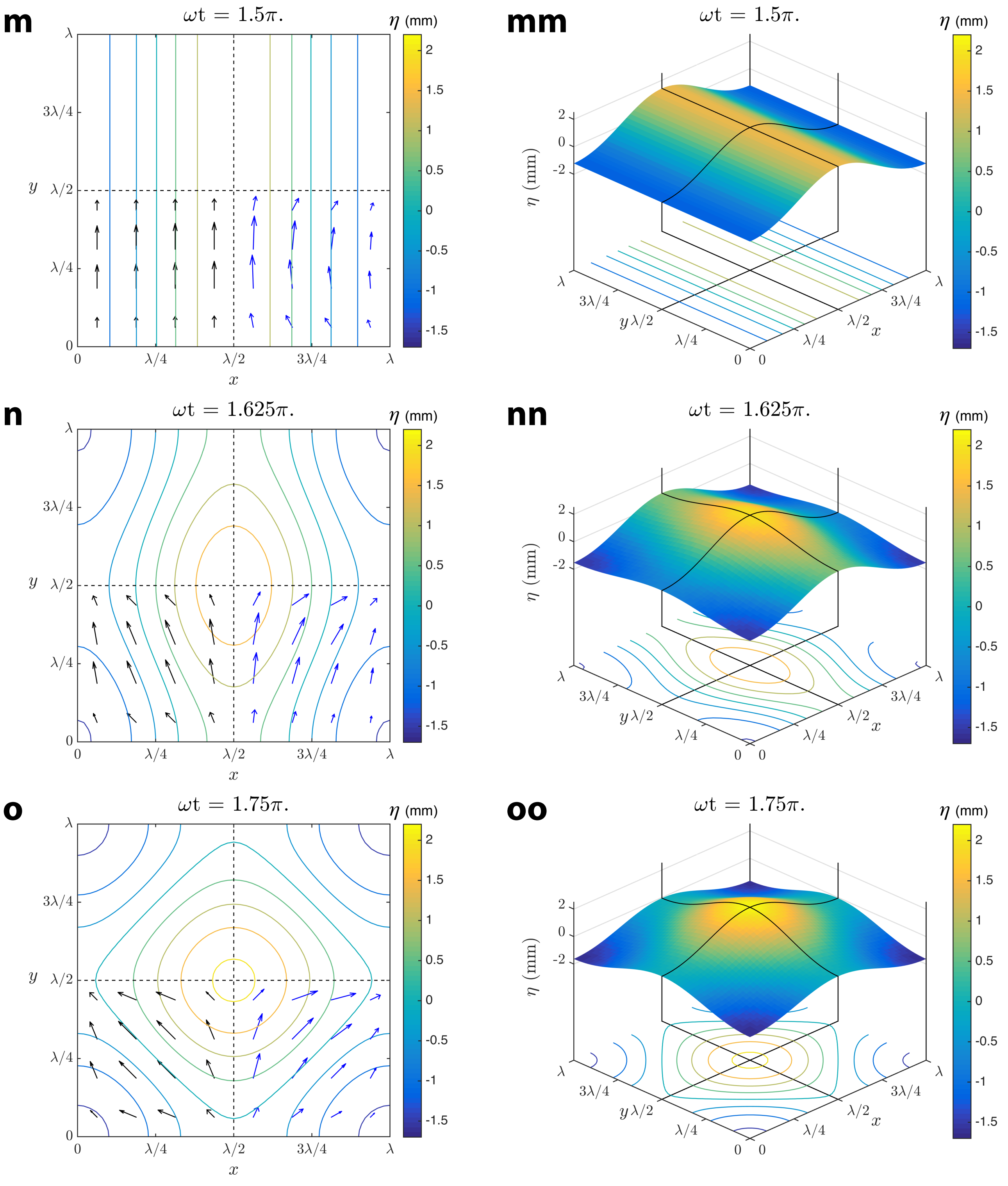

m
ωt = 1.5π.
η (mm)
mm
ωt = 1.5π.
η (mm)
n
ωt = 1.625π.
η (mm)
nn
ωt = 1.625π.
η (mm)
o
ωt = 1.75π.
η (mm)
oo
ωt = 1.75π.
η (mm)
x
y
λ/4
λ/2
3λ/4
λ

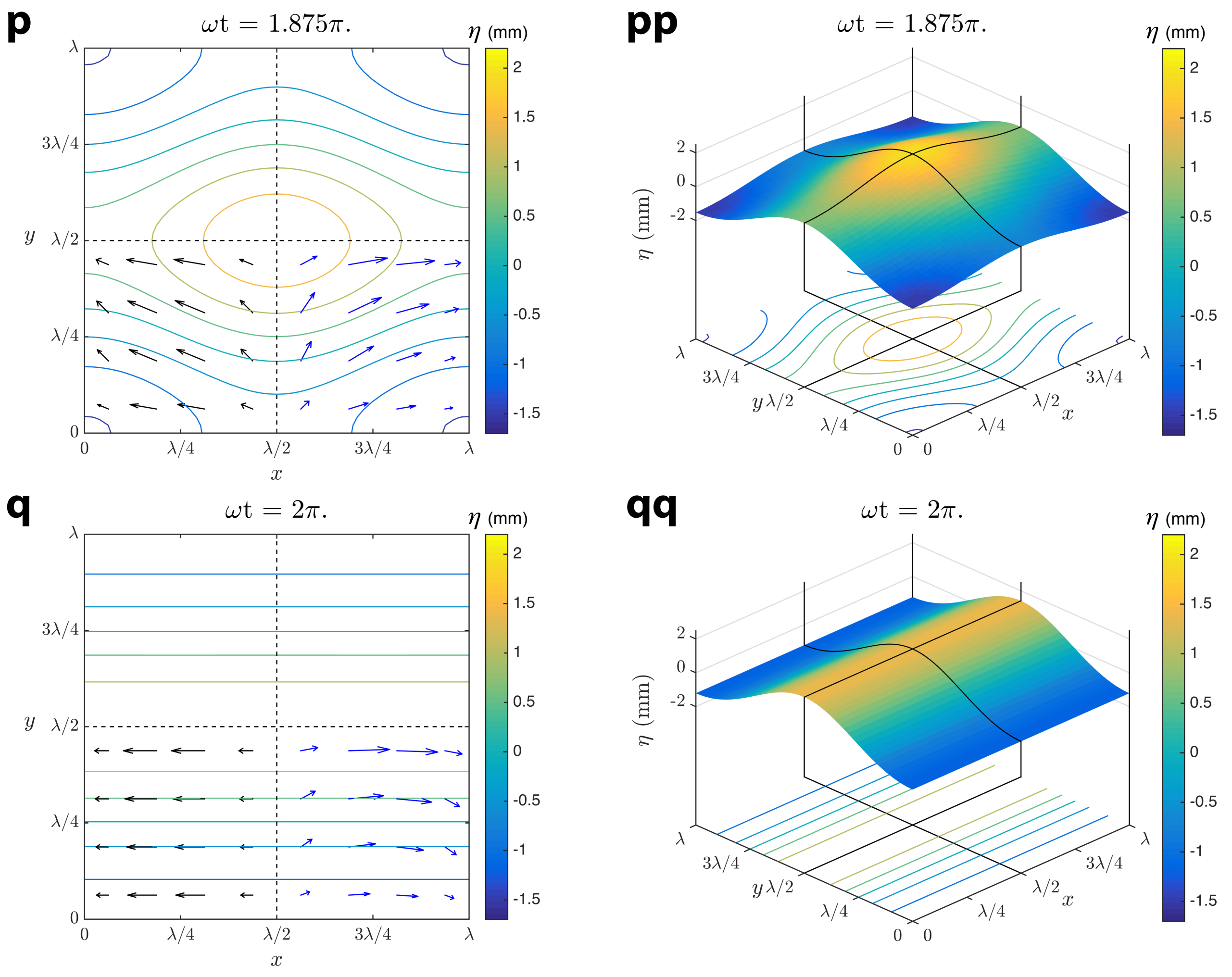

Supplementary Figure 3: Modelled surface elevation and velocities for two orthogonal standing surface waves phase shifted by $\phi = \pi/2$. (see Movies 3 and 4) Panels are shown every 1/16th of a cycle from $\omega = 0$ to $\omega t = 2\pi$ on a $\lambda \times \lambda$ box, i.e. 2×2 unit cells. **a-p**: contours of constant η. Arrows in the lower left unit cell (black) show the potential velocity $\boldsymbol{u}^P$. Arrows in the lower right (blue) show the potential velocity $\boldsymbol{u}^P$ plus the stationary rotation $\boldsymbol{u}^R$. **aa-pp**: surface and contour plots of η at the same times. Note the counterrotating surfaces in neighboring unit cells and the discrete jumps of the peak surface displacement from one corner to the next in each unit cell. The corners of each unit cell are antinodes, and the center of each is a node. The velocity tends to run along contours of constant η. Within each unit cell, the rotations associated with the surface wave, the small particle gyrations, and the particle drift orbits are all in the same direction.
