# Supplementary figures and images for: Wave-based liquid-interface metamaterials
Source: Nat Commun. 2017 Feb 9;8:14325. doi: 10.1038/ncomms14325 (PMC5311468; doi:10.1038/ncomms14325)

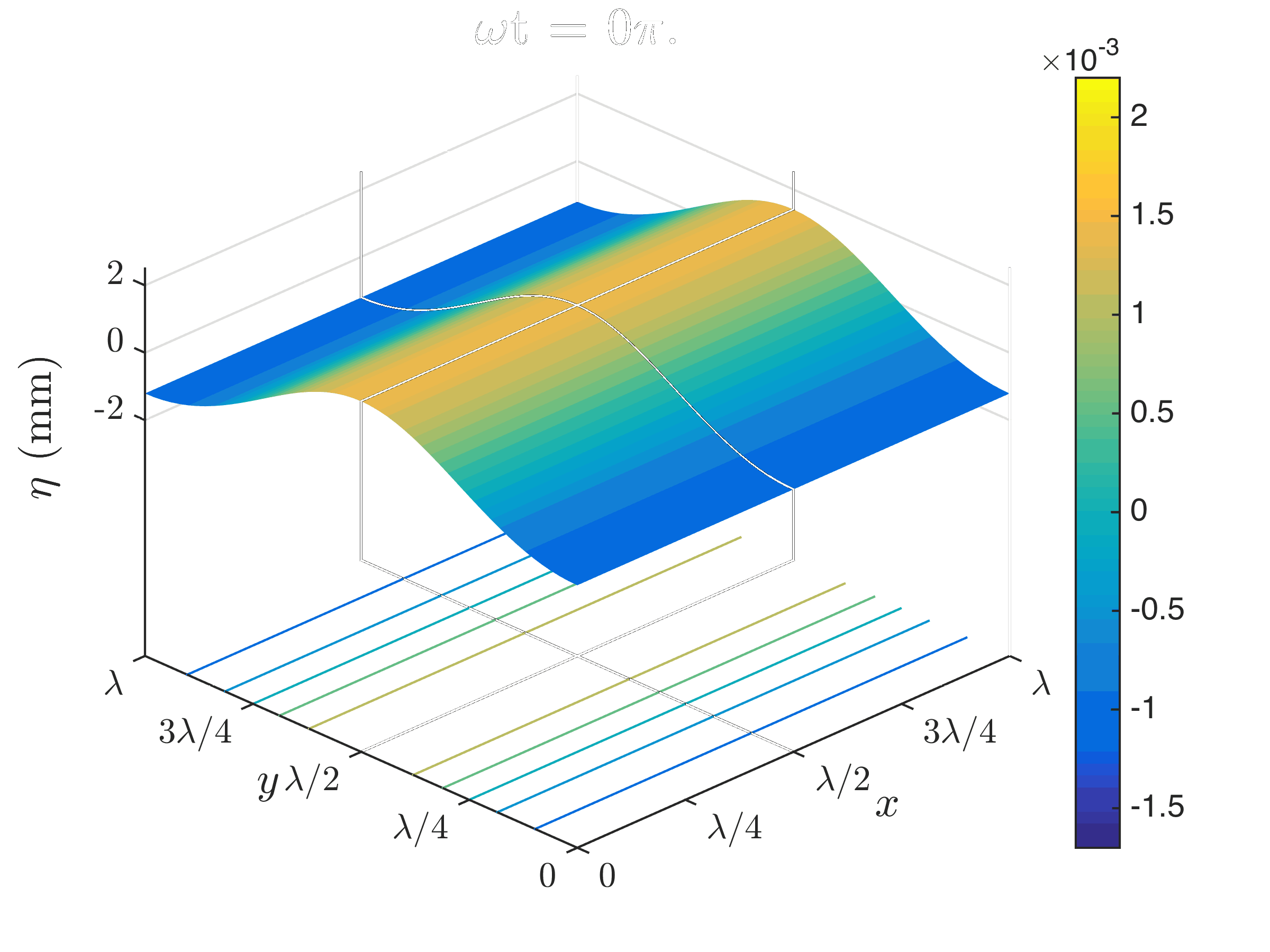

Supplement: Supplementary Movie 3 — Theoretically simulated surface and contour plots of surface elevation on a 2x2 unit cell box. Time scale is one wave period. [file ncomms14325-s4.tif]

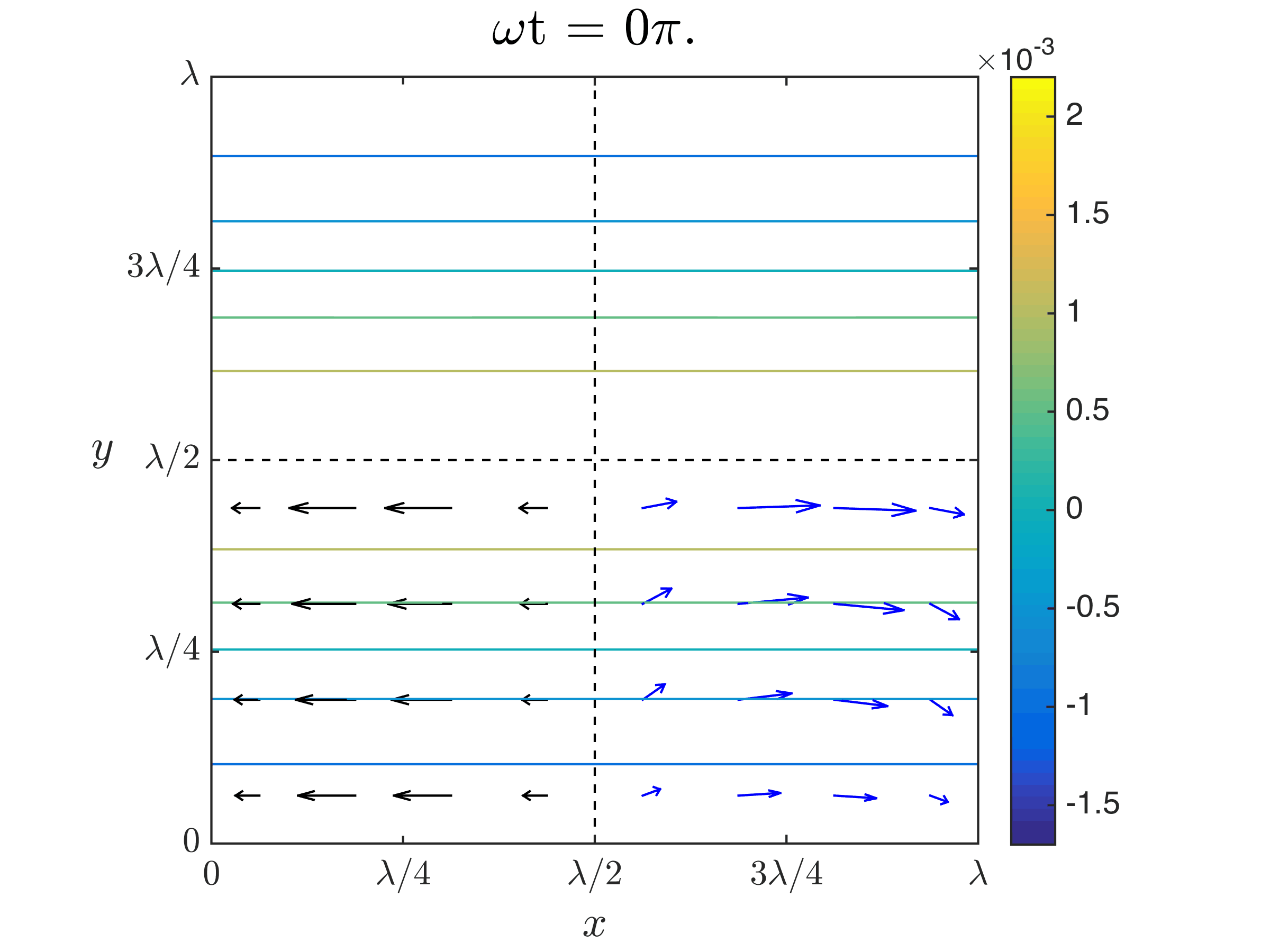

Supplement: Supplementary Movie 4 — Theoretically simulated contours of constant surface elevation and vectors of the potential velocity on a 2×2 unit cell box. Time scale is one wave period. [file ncomms14325-s5.tif]
